# A Long Short-Term Memory Biomarker-Based Prediction Framework for Alzheimer’s Disease

**DOI:** 10.3390/s22041475

**Published:** 2022-02-14

**Authors:** Anza Aqeel, Ali Hassan, Muhammad Attique Khan, Saad Rehman, Usman Tariq, Seifedine Kadry, Arnab Majumdar, Orawit Thinnukool

**Affiliations:** 1 Department of Computer & Software Engineering, CEME, NUST, Islamabad 44800, Pakistan; anzaaqeel@hotmail.com (A.A.); alihassan@ce.ceme.edu.pk (A.H.); 2 Department of Computer Engineering, HITEC University, Taxila 47080, Pakistan; attique@ciitwah.edu.pk (M.A.K.); saad.rehman@hitecuni.edu.pk (S.R.); 3 College of Computer Engineering and Science, Prince Sattam Bin Abdulaziz University, Al-Kharaj 16242, Saudi Arabia; u.tariq@psau.edu.sa; 4 Department of Applied Data Science, Noroff University College, 4608 Kristiansand, Norway; skadry@gmail.com; 5 Department of Civil Engineering, Imperial College London, London SW7 2AZ, UK; a.majumdar@imperial.ac.uk; 6 College of Arts, Media and Technology, Chiang Mai University, Chiang Mai 50200, Thailand

**Keywords:** Alzheimer’s, long short-term memory, artificial neural network, machine learning

## Abstract

The early prediction of Alzheimer’s disease (AD) can be vital for the endurance of patients and establishes as an accommodating and facilitative factor for specialists. The proposed work presents a robotized predictive structure, dependent on machine learning (ML) methods for the forecast of AD. Neuropsychological measures (NM) and magnetic resonance imaging (MRI) biomarkers are deduced and passed on to a recurrent neural network (RNN). In the RNN, we have used long short-term memory (LSTM), and the proposed model will predict the biomarkers (feature vectors) of patients after 6, 12, 21 18, 24, and 36 months. These predicted biomarkers will go through fully connected neural network layers. The NN layers will then predict whether these RNN-predicted biomarkers belong to an AD patient or a patient with a mild cognitive impairment (MCI). The developed methodology has been tried on an openly available informational dataset (ADNI) and accomplished an accuracy of 88.24%, which is superior to the next-best available algorithms.

## 1. Introduction

Alzheimer’s disease (AD) is a developing and unchangeable degenerative cerebral illness defined by recognition failure and psychological disability. According to [1] the Alzheimer’s Association (2019), in recent times, approximately, 90 million people are admitted to hospitals due to AD worldwide. According to the study by [2,3], the estimated number of deaths due to Alzheimer’s disease will reach 300 million by the year 2050. There is still no specific treatment that has been developed which can cure or stop this disease and save the patient’s life. A few treatments are used by medical practitioners that can delay the rate of AD when detected at the initial stages. 

Mild cognitive impairment (MCI) [4] impacts memory, language, thinking, and judgment in a way that is more noticeable than conventional age-related changes. If an individual has minor intellectual impairment, he/she may realize that their memory or intellectual ability has “slipped.” People close to the person may see more significant change. Regardless, these changes are usually not extensive enough to interfere with day-by-day life and typical activities. An impaired mental capacity may become worse over time, causing dementia or other neurological conditions. In any case, some patients with minor impairments do not become worse, and some even improve in the long term.

Mild cognitive impairment is one of the preliminary signs of AD [5]. Recent neuroimaging technology is widely employed to identify a few essential biomarkers in the brain for the diagnosis of brain tumors [6]. Similarly, this research brings about an automated system in which NM and MRI values are calculated as important biomarkers to detect AD in the human brain.

Although there is no proven biomarker for the correct or accurate prediction of whether a patient will progress from MCI to AD, multiple approaches have been adapted in different studies to predict the growth of disease. Studies that use different prediction models to predict the growth of disease mostly depend on clinical modalities like magnetic resonance imaging scans [7,8], neuropsychological measures, computerized tomography scans [9,10], diffusion tensor imaging, CSF biomarkers, and positron emission tomography [11,12]. For example, in a study conducted by [13], the authors used a combination of cerebrospinal fluid, fluorodeoxyglucose–positron emission tomography, and MRI biomarkers for the classification of patients who will progress to AD from MCI (stable state). Nevertheless, studies about reducing the growth of disease over time are limited. In the medical domain, most devices are employed for public use, and it is easy to access medical data from several sources [14,15]. These sources can be MRI and CT scans that are longitudinally obtained [16,17]. Conventional methods are mostly used for the analysis of knowledge extraction among several variables (quantities). ML algorithms have provided much help in the prediction of AD [18]. By using algorithmic techniques, it has become possible to gather relevant input data, and to perform some algorithms and generate output, which can be a prediction of the highlighted disease. These techniques can also be beneficial for finding the relationships within the input data, and can help in the early prediction of diseases.

MCI is an early-stage disease which is generally not harmful, and patients who have this disease are likely to function at normal levels. A prediction model for predicting which patients will progress from MCI to AD could help with early diagnosis and an early cure or treatment of the disease. Thus, the significance of the study is also the same as that which will contribute to the question of whether a patient’s MCI will progress into AD or not. Various biological markers may be used in the prediction of a patient’s disease by their doctor. Similarly, the ML model will forecast the progression of disease using some of the key biomarkers. The NM and MRI values of the patients are biomarkers, like features in ML. Based on these biomarkers, the ML model will create a prediction about whether the patient will progress to AD in the next three years or not. This process is helpful for doctors and clinical staff for speeding up the prediction process. Therefore, a need exists to formulate the question: “To what extent can a classifier predict the progression of subjects from MCI to AD, based on NM and MRI biomarkers?”.

Many techniques have been introduced within the research of computer vision in the medical domain, such as fuzzy clustering [19], prostate zonal segmentation [20], and a few more [21]. Recently, deep learning has had a great impact in the area of medical diagnoses [22], such as for skin cancer [23,24,25], brain tumors [26], stomach [27], COVID-19 [28], person re-identification [29] and a few more [30]. The projected work proposes a ML model for the prediction of progression to Alzheimer’s disease using long short-term memory in a recurrent neural network. Deep learning has revolutionized the area of image and video processing and computer vision [31]. In the proposed model, the NM and MRI biomarkers (feature vectors) are computed and passed to the RNN. In the RNN, we use LSTM, which has never been used in the context of AD before. This is the gap that as researchers, we are trying to fill in order to understand the more intricate and complex particularities of AD. This model will predict the biomarkers (feature vectors) of patients after 6, 12, 18, 24, and 36 months. Then, these predicted biomarkers be passed through a fully connected neural network model, a multi-layer perceptron, also known as a convolutional neural network. It will predict whether these RNN-predicted biomarkers belong to an AD patient or an MCI patient. If the predicted values provided by RNN lay within the range of values depicting whether a person will have AD in the future, then the patient’s record can updated, and whether they are more likely to evolve from MCI to AD in the future will also be predicted by CNN.

Contributions of this research are as follows:

- Projecting future clinical variations in biomarker values, only utilizing initial/benchmark information/data.
- RNN is performed to predict biomarker values and then rankings, followed by a fully connected neural network model (multi-layer perceptron) for classification, in which an accuracy of 88.24% is achieved.
- Identifying the strongest indicators of transformation in unimodal and multimodal settings.
- This study is significant for medical practitioners and health care workers in the early prediction and detection of Alzheimer’s disease; moreover, future researchers can adopt this model as a basis for their studies to further contribute to the development of algorithms for predicting Alzheimer’s disease in the future.
- This study also serves as a training tool for medical institutions to educate and train their students regarding the early prediction and development of Alzheimer’s disease.

The paper is structured as follows: Section 2 involves the related literature, Section 3 portrays the adapted methodology, Section 4 evaluates the outcomes and provides a short comparison with recently distributed work. Section 5 concludes the paper.

## 2. Literature Review

Recently, deep learning has shown improved performance for medical applications [32,33] such as breast cancer [34], retinopathy [35], COVID-19 [36], and many more [37]. The need to foresee the advancement to AD from MCI is consistently important to help treat this illness in its initial phase. It is critical to understand how this illness develops after some time, and for a better understanding it is imperative to know of related irregularities that happen in the cerebrum. Familiarity with these irregularities is important to choose the attributes that predict progression to AD. Recently, numerous ML methods have been proposed for AD forecasting. The vast majority of strategies rely upon the quantity of features, while not many of them are dependent on clinical features. In [3], a hybrid approach for the analysis of the hippocampus using MRI in AD patients was presented. They used a pre-trained DenseNet architecture for the intensity and shape of features. Then, they computed and trained high-level features by combining RNN, and then performed a final classification. The “Alzheimer’s Disease Neuroimaging Initiative” Database (www.adni.loni.edu (accessed on 22 December 2021)) was used for experimental analysis and showed improved results compared to existing techniques. Basheera & Ram [38] proposed a deep learning-based methodology for Alzheimer’s disease classification. They used MRI types such as T2 weighted volumes, which included 635 MRIs of AD patients and 548 MCI patients. They extracted gray matter features from MRI voxels and passed these to a convolutional neural network. Then, they enhanced brain voxels using a Gaussian filter, and removed the irrelevant masses by skull stripping. Later, segmentation was performed by component analysis, and they passed the output image to CNN for the final prediction. Overall, 90% clinical accuracy was attained, which was better than existing techniques. Basheera & Ram [39] presented a hybrid clustering and CNN model for the prediction of AD, MCI, and CN. They performed skull stripping at the initial stage and improved enhancement via a Gaussian filter. Then, they combined K-means and expectation maximization (EM) methods, and performed segmentation. The fragmented pictures were passed to the CNN model for feature extraction and the final prediction. The authors of [40] presented a multitasking ML model for AD prediction. The regression model defined each task separately and predicted a cognitive score. This interaction was produced for all tests, and towards the end, a relationship was elucidated among them. Finally, the multiple task scores performed were passed to a slope boosting piece for better forecasting. Thus, this interaction assisted in eliminating the irrelevant features for a better prediction. Lei [41] presented an AD prediction framework using longitudinal data. In this study, they calculated the clinical score as a feature value. Then, they performed feature selection via a corr-entropy approach. Next, the selected features were encoded in a deep polynomial network. Finally, the prediction was performed through a support vector machine. The ADNI dataset was used for the experiment and attained an impressive performance. Furthermore, a biomarker-based approach was also presented by [42], which shows its performance for the correct prediction of patients’ development towards AD from normal levels. In this work, NM and MRI biomarkers are extracted as feature vectors, and perform learning through autoregressive modeling.

## 3. Proposed Methodology

**Figure 1:**
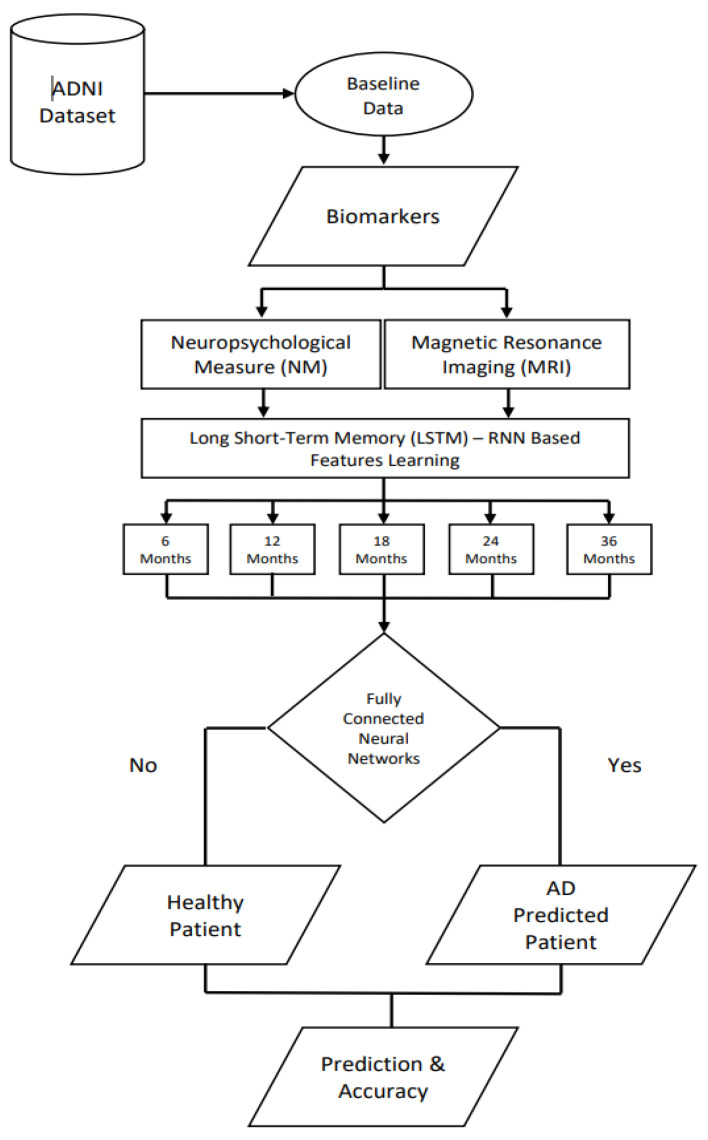
The proposed architecture of Alzheimer’s disease’s prediction.

The proposed work comprised of deep learning and biomarker techniques for AD prediction. The proposed framework consists of few primary steps, as shown in Figure 1, such as original baseline data, biomarker extraction as feature vectors, learning features through RNN type name LSTM, and prediction via MLP. Before prediction via CNN, the recurrent neural network features were updated based on the monthly patient record. The details of each listed step in Figure 1 are shown below. 

### 3.1. ADNI Dataset

For AD, the most mainstream sample set is the ADNI, which was used in this work for validation of the projected framework. In this dataset, 805 subjects were included. These cases were gauge MRI T1-weighted (T1w) information. The ADNI dataset (www.adni.loni.edu (accessed on 22 December 2021)) incorporates the positive biomarkers of patients after every 6, 12, 18, 24, and 36 months from the standard. The primary objective of utilizing this dataset was to inspect whether MRI, positron emission tomography, organic markers, and clinical evaluations can be combined to encompass the improvement of MCI and early findings of Alzheimer’s disease.

### 3.2. RNN-LSTM

**Figure 2:**
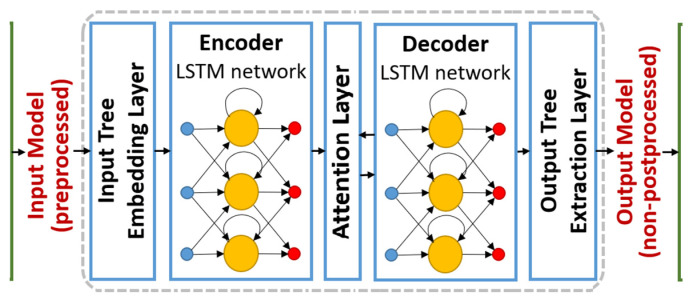
The architecture of the LSTM model (www.modelling-languages.com/lstm-neural-network-model-transformations/ (accessed on 22 December 2021)).

In this work, the NM and MRI biomarkers were extracted as feature vectors. A feature vector is a vector containing multiple elements about an object. The purpose of the LSTM model is to predict the future feature vectors (biomarkers) of the patient. The biomarker value changes as per the patient’s condition. This model can predict what condition the patient’s brain will be in after 6, 12, 18, 24, and 36 months. A proposed algorithm was trained using 805 patients’ data, as mentioned in the above section. We provided baseline (0 months or the first NM + MRI test of the patient), NM, and MRI biomarkers (feature vectors) as inputs in the model. We trained our model on the biomarkers of patients after 6, 12, 18, 24, and 36 months, as provided in the dataset. Mathematically, this model was formulated as follows: the standard engineering of the LSTM network comprises an info layer, a repetitive LSTM layer, and a yield layer. The data layer is linked with the LSTM layer, as demonstrated in Figure 2.

The tedious relations in the LSTM layer are between cell input units and yield units, inputs, yield entryways, and disregard doorways. The cell yield units are related to the yield layer. The number of limits, *P*, in a standard LSTM network containing one cell in each memory block, can be calculated as:

(1) $$P=l_{c}\times l_{c}\times 4+l_{i}\times l_{c}\times 4+l_{c}\times l_{o}+l_{c}\times 3$$

where $l_{c}$ means the memory cells, $l_{i}$ is the number of information components, and $l_{o}$ is the quantity of yield units. The time intricacy of the LSTM learning network with a stochastic inclination plummet (SGD) advancement strategy is *O*(1). Similarly, intricacy per time step is *O*(*P*). Learning time is affected by the factor $l_{c}$ × ($l_{c}$ + $l_{o}$), when the quantity of information sources is generally few. The LSTM learning model becomes costly in terms of intricacy, when the quantity of yields and memory cells are large. The accompanying conditions charts the organization unit enactments iteratively from *z* = 1 to *Z*, in order to ascertain predictions from an information arrangement $a=\left(a_{1},\;\ldots ,\;a_{Z}\right)$ to $b=\left(b_{1},\;\ldots ,\;b_{Z}\right)$:

(2) $$g_{z}=\sigma \left(M_{ga}a_{z}+M_{gk}k_{z-1}+M_{gj}j_{z-1}+bias_{g}\right)$$

(3) $$h_{z}=\sigma \left(M_{ha}a_{z}+M_{kh}k_{z-1}+M_{jh}j_{z-1}+bias_{h}\right)$$

(4) $$j_{z}=h_{z}⊙j_{z-1}+g_{z}⊙s\left(M_{ja}a_{z}+M_{jk}k_{z-1}+bias_{j}\right)$$

(5) $$o_{z}=\sigma \left(M_{oa}a_{z}+M_{ok}k_{z-1}+M_{oj}j_{z-1}+bias_{o}\right)$$

(6) $$k_{z}=o_{z}⊙t\left(j_{z}\right)$$

(7) $$b_{z}=M_{bk}k_{z}+bias_{b}$$

where *M* is the weight lattices (e.g., $M_{ga}$ is the weight grid from input door to yield entryway), *σ* means the strategic sigmoid capacity, *g*, *h*, *j*, and *o* are the information door, neglect door, cell enactment vector, and yield door, *k* is the cell actuation vector, ⊙ computes the results of the vectors, and s and t are the initiation capacities (mostly tanh) for cell information and yield. The equations for the last LSTM model with both repetitive and non-intermittent projection layers are depicted beneath:

(8) $$g_{z}=\sigma \left(M_{ga}a_{z}+M_{gx}x_{z-1}+M_{gj}j_{z-1}+bias_{g}\right)$$

(9) $$h_{z}=\sigma \left(M_{ha}a_{z}+M_{xh}x_{z-1}+M_{jh}j_{z-1}+bias_{h}\right)$$

(10) $$j_{z}=h_{z}⊙j_{z-1}+g_{z}⊙s\left(M_{ja}a_{z}+M_{jx}x_{z-1}+bias_{j}\right)$$

(11) $$o_{z}=\sigma \left(M_{oa}a_{z}+M_{ox}x_{z-1}+M_{oj}j_{z-1}+bias_{o}\right)$$

(12) $$k_{z}=o_{z}⊙t\left(j_{z}\right)$$

(13) $$x_{z}=M_{xk}k_{z}$$

(14) $$y_{z}=M_{yk}y_{z}$$

(15) $$b_{z}=M_{bx}x_{z}+M_{by}y_{z}+bias_{b}$$

where x denotes the recurrent and y non-recurrent unit activation functions. 

### 3.3. Multi-Layer Perceptron (MLP) 

MLP is a type of ANN. The term MLP is used vaguely, and at times freely, to any feedforward ANN, now and again stringently alluding to networks made out of distinctive layers of perceptron. MLP contains 3 layers: a data layer, a hidden layer, and an output layer. As well as the information center points, each center point is a neuron that uses nonlinear activation. MLP utilizes a directed-learning procedure returned to spread for planning. Its various layers and non-direct establishments perceive MLP from a straight perceptron. It can perceive data that is not straightforwardly detachable. In case a multi-layer perceptron has an abrupt authorization work in all neurons, that is, a straight limit that maps the weights to the output of each neuron, direct polynomial numbers show that many layers can be diminished to a 2-layer I/O model. In MLPs, a couple of neurons use nonlinear inception work that was made to show the repeat of movement prospects or ending of neurons.

**Figure 3:**
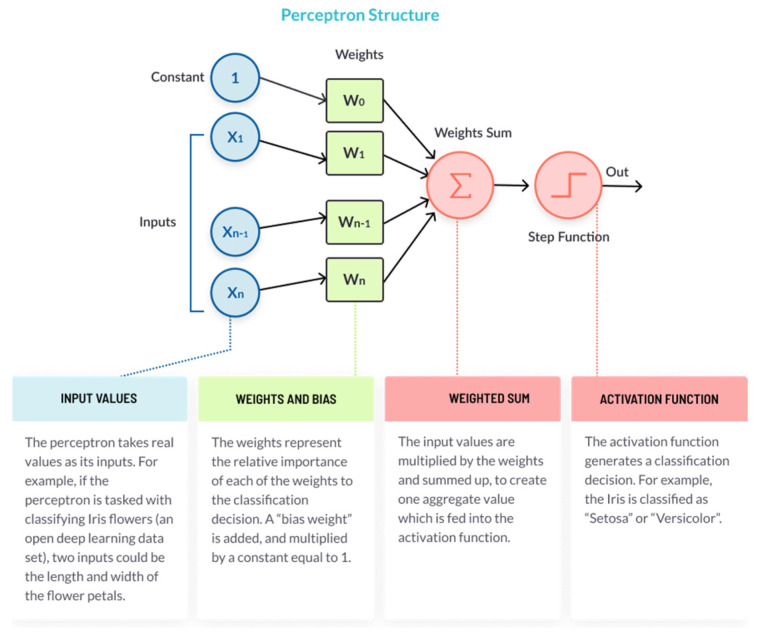
Perceptron structure.

In later developments, a rectifier linear unit (RELU) is used as one of the likely ways to deal with and conquer the numerical issues determined to have the sigmoid from time to time. The multi-layer perceptron (MLP) contains, in any event, three layers (an information and a yield layer, with one mystery layer) of nonlinear-starting center points. Since multi-layer perceptrons are related, each center point in one layer interfaces with a particular weight W_ij_ to every center point in another layer. The phrase “multi-layer perceptron” does not imply a single perceptron that has different layers. Instead, it contains various perceptrons that are composed into layers. An alternative name is a “multi-layer perceptron network”. Furthermore, MLP “perceptrons” are not perceptrons in the strictest possible sense. Authentic perceptrons are a formally unprecedented instance of fake neurons that use a cut-off order limit, for example, the Heaviside step work. MLP perceptrons can utilize self-assertive actuation capacities. A genuine perceptron performs in a two-fold arrangement, whereas an MLP neuron is free to either perform in order or relapse, contingent on its actuation work (see Figure 3).

The expression “multi-layer perceptron” was later applied without regard to the nature of the hubs/layers, which can be made out of discretionarily characterized counterfeit neurons and not perceptrons explicitly. This translation dodges the loosening of the meaning of “perceptron” to mean a counterfeit neuron. The perceptron, or neuron in a neural organization, has a basic yet sharp construction. It comprises four sections:

- It takes the data sources, duplicates them by their loads, and calculates their total
- It adds an inclination factor, the number 1 duplicated by a weight
- It feeds the aggregate through the enactment work
- The result is the perceptron yield

Despite the fact that multi-layer perceptrons and neural organizations are basically the same thing, a couple of fixes need to be added before a multi-layer perceptron becomes a full neural organization. These are back spread, hyper boundaries, and advanced constructions.

## 4. Results and Discussion

The projected system is presented as numerical and tabular results. The validation of the proposed technique was performed using the ADNI dataset as previously explained. The patients’ data were collected after 6, 12, 18, 24, and 36 months. Based on this data, LSTM was trained and output was passed to a multi-layer perceptron network (artificial neural network). This classifier predicts MCI and AD patients based on their monthly values. The performance of this method was calculated by accuracy measures and a false negative rate. Furthermore, the confusion matrix was also produced for verification of the proposed results. Then, the proposed results were compared with previous techniques at the end of this section. All results were computed through a 70:30 approach where cross-validation was 5-Fold. Python was used for implementing this approach on a Corei7 personal computer with 16GB of RAM. 

### 4.1. Results

The proposed forecast results are outlined regarding exactness esteems, root mean square mistakes, and connection coefficients. The outcomes were determined using various mixes of hidden layers. A hidden layer is situated between the information and yield of the calculation, where the capacity applies loads to the data sources, and guides them through initiation work as the yield. Thus, hidden layers perform nonlinear changes to the information sources put into the organization. Hidden layers fluctuate depending on the capacity of the neural organization, and comparatively, layers may change depending on their related loads.

Hidden layers consider the capacity of a neural organization to be separated into explicit changes in the information. Each hidden layer work is specific to deliver a characterized yield. For instance, hidden layer works that are used to recognize natural eyes and ears might be utilized by resulting related layers to distinguish faces in pictures. While the capacity to distinguish eyes alone is not adequate to freely perceive objects, these layers can work together inside a neural organization.

**Figure 4:**
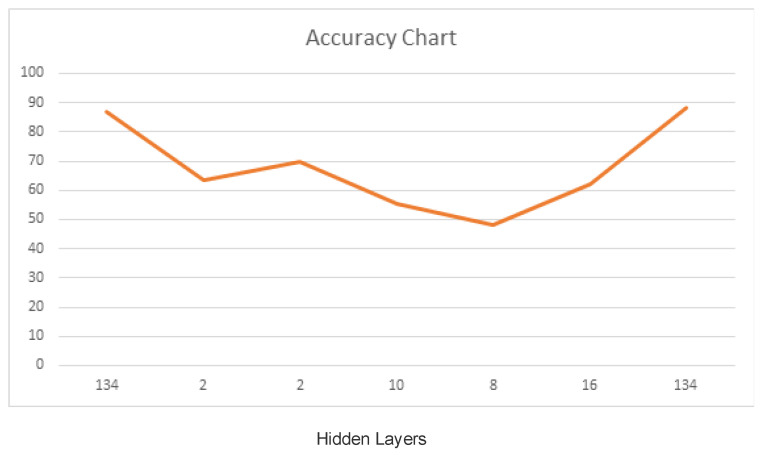
Proposed AD Prediction Results in Terms of Accuracy Value.

The accuracy chart (Figure 4) depicts a clear illustration of results when the output model results from the recurrent neural networks (LSTM) were passed through a fully connected neural network model, MLP and ANN. Hidden layers play important roles in determining the accuracy of the predicted results, that is, whether these results are accurate or not. The x-axis shows the number of hidden layers used, while the y-axis states the accuracy results. The result with 88.24% accuracy was chosen to be the best result obtained, and this result was obtained using a 5-Fold cross-validation model. K-fold cross CV is a calculation where a given instructive list is separated into a K number of zones/folds, where each wrinkle is used as a testing set in the long run. We should take the circumstance of 5-Fold cross-validation (K = 5). 

Table 1 indisputably depicts the yield of results gained using different mixes of characteristics for hidden layers, learning rates, and energy. The related layers of the artificial neural network was used for gathering the yields from the LSTM model, which contains the expected biomarkers of the patient from check-up to a year and a half. Specifically, the learning rate is a configurable hyper-limit used in the readiness of neural associations that has a particular value, usually between 0.0 and 1.0. The learning rate controls how quickly the model is changed in accordance with the issue. More unassuming learning rates require additional preparation time, especially when more unobtrusive changes are made to the model at each update; however, greater learning rates achieve quick changes and require less preparation time. A learning rate that is too big can cause the model to unnecessarily combine obtrusive changes, although a learning rate that is too small can cause the connection to slow down.

Learning rate and force was often changed during various endeavors in order to acquire outcomes that provided the most extreme exactness when 134 hidden layers were added to the model, using a learning pace of 0.3 and energy of 0.2, while keeping cross-validation folds at 5 provided the best outcomes. On sequence 1, there were 134 hidden layers, yet the model used 10-fold cross-validations which provided a precision of 86.97%. When folds were reduced to 5, the model showed a great accuracy of 88.24%.

**Figure 5:**
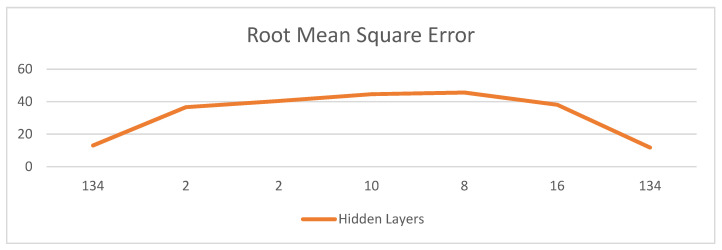
The root mean square error.

The root mean square error is a reliably used measure of the degree of the separation between values (with respect to both tests and individuals) expected by a model or validator and the attributes that are observed (see Figure 5). The root mean square deviation focuses on the square base of the second model, indicating the separation between the expected attributes and actual attributes or the quadratic mean of these capabilities. These deviations are called remains/residuals when the computations are performed using a staggering model for examination, and are called goofs (or presumption bungles) when dealt with out-of-test. The RMSD serves to characterize the level of variation in the model. 

Table 2 shows the results of the RMSE when using different combinations of hidden layers and other variables. The minimum value of the root mean square error reflects the maximum accuracy and efficiency of our model. Several different values of components were incorporated to achieve the minimum error (that is, maximum accuracy) output for the model for predicting the number of patients who progressed from MCI to AD. The two closest combinations were when the number of hidden layers was 134, with cross-fold validations of 5 and 10. In the case of 10-fold cross-validation, the RMSE was almost 13%, which means the accuracy of the model was 87%; however, when the number of folds used in cross-validation decreased to 5, the RMSE decreased and was computed to be 12%, which gave sufficiently good output with an accuracy of 88.24%. Other combinations of hidden layers proved to be less effective in computing the accuracy of the system in predicting the number of patients who would progress from MCI to AD.

**Figure 6:**
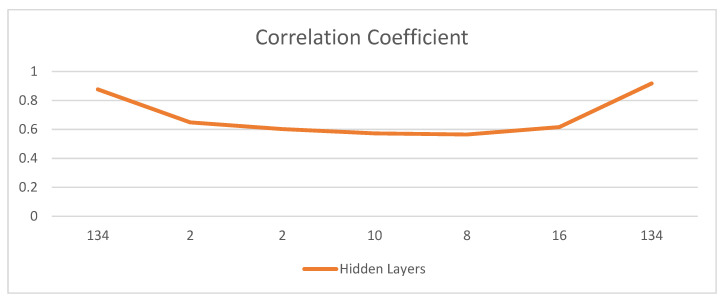
The correlation coefficient.

Correlation coefficients represent an authentic level of the strength of the link between the overall advancements of two variables (see Figure 6). Its characteristics range between −1.0 and 1.0. A computed number higher than 1.0 or less than −1.0 suggests that there was an error in the assessment of a relationship. An association of −1.0 shows an negative relationship, while an association of 1.0 shows a positive relationship.

### 4.2. Analysis

A comprehensive analysis is formulated in this section which explains changes in the performance of each biomarker combination with different hidden layers. The proposed AD prediction architecture used two biomarkers, NM and MRI. Then, RNN-type LSTM-based features are learned and passed to a fully connected neural network layer using Python framework. The findings are tabulated in Table 1 in the form of accuracy/precision values, and attained the best accuracy of 88.24% with biomarker combinations of 4 NM and 35 MRI. Furthermore, the RMSE values are also shown in Figure 5 and Table 2, followed by the values of the obtained correlation coefficients while using different numbers of hidden layers.

**Figure 7:**
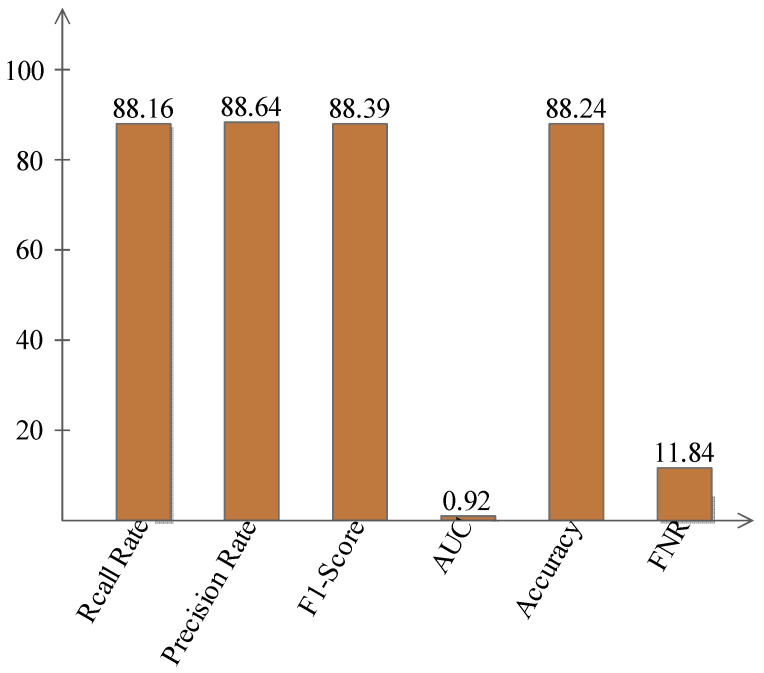
The performance measures computed for the best accuracy value.

In Table 1, the best-noted accuracy was 88.24%; other computed measures such as recall rate was 88.16, the prevision rate was 88.64, the F1-Score was 88.39, and the FNR value was 11.84%. The AUC was also computed with a value of 0.92. These values are plotted in Figure 7. 

Table 3 shows a clear comparison of the results computed through different algorithms and techniques by other authors by using mild cognitive impairment (MCI) samples. In [43], the authors presented a method for AD prediction and achieved an accuracy of 79%. In [44], the researchers achieved an accuracy of 73.95% using an MRI + NM biomarker approach. In [45], the authors used both NM and MRI biomarkers for predictions in their model and achieved an accuracy of 86.6%. 

In [46], the authors created a model using a dataset of 320 patients with 2-year follow-ups and achieved an accuracy of 80.1%. In the proposed approach, we utilize MRI and NM biomarkers, and the model learned using RNN-LSTM, then evaluated the data via a fully connected NN layer for classification. The quantity of hidden layers used for this model was 134, with a learning rate of 0.3 and momentum set as 0.2, with 5-fold cross-validation and a correlation coefficient of 0.9172, and achieved an accuracy of 88.24% with the root mean square error as 0.117, which is an improvement compared to these previous techniques. 

## 5. Conclusions

The current experiment aimed to research the degree to which it is feasible to predict a patient’s progression from MCI to AD, and this current models comparison with previous models. The current investigation has demonstrated that the recurrent neural network (LSTM) performed better than other previously utilized classifiers. The most fascinating finding in the current investigation was that the model can anticipate illness progression. However, the model could be improved if more extensive follow-up times indicating progression were used. Despite the fact that the model lacks down-to-earth applications, the current investigation makes a few essential commitments to the field of AD expectation. This is a key experiment that has used a multi-layer perceptron to predict illness progression. The current examination offers some knowledge into the significance of choosing the correct follow-ups, which adds to progress in discovering better models to use in the future. Improved exactness rates accomplished by using an RNN in this experiment demonstrates that these are truly outstanding and precise predictions of progression from MCI to AD. In the future, dynamic graph convolution [47] and multi-view feature learning [48] techniques shall be consider. 

## Figures and Tables

**Table 1:** Accuracy results.

Trials	Hidden Layers	Learning Rate	Momentum	Cross-Validation Folds	Correlation Coefficient	RMSError	Accuracy
1	134	0.3	0.2	10	0.8767	0.13	86.97
2	2	0.1	0.1	10	0.6487	0.37	63.36
3	2	0.3	0.2	5	0.602	0.40	70
4	10	0.3	0.2	5	0.5722	0.45	55.37
5	8	0.3	0.2	10	0.565	0.46	48
6	2, 4, 8, 16	0.3	0.2	10	0.6163	0.38	61.92
7	134	0.3	0.2	5	0.9172	0.12	88.24

**Table 2:** The root mean square error (RMSE).

Trials	Hidden Layers	Root Mean Square Error
1	134 (10-Fold Cross-Validations)	0.13
2	2	0.37
3	2	0.40
4	10	0.45
5	8	0.46
6	2, 4, 8, 16	0.38
7	134 (5-Fold Cross-Validations)	0.12

**Table 3:** A comparison of results between existing techniques.

Results Comparison				
Author	Biomarkers	Sample Size	Duration (Years)	Accuracy/Precision (%)
Minhas et al. (2017) [42]	NM & MRI	54 MCIp & 65 MCIs	2	84.29
Minhas et al. (2017) [42]	NM	37 MCIp & 65 MCIs	3	83.26
Arco et al. (2016) [44]	MRI & NM	73 MCIp & 61 MCIs	1	73.95
Albright et al. (2019) [45]	NM & MRI	110 Patients	2	86.6
Our Results	NM & MRI	167 MCIp & 100 MCIs	3	88.24

## Data Availability

Not applicable.

## Abbreviations

ML	Machine Learning
AD	Alzheimer’s Disease
NM	Neuropsychological Measures
MRI	Magnetic Resonance Imaging
RNN	Recurrent Neural Network
LSTM	Long Short-Term Memory
MCI	Mild Cognitive Impairment
CNN	Convolutional Neural Network
MLP	Multi-Layer Perceptron
ANN	Artificial Neural Network
